# Characterization of Breed Specific Differences in Spermatozoal Transcriptomes of Sheep in Australia

**DOI:** 10.3390/genes12020203

**Published:** 2021-01-30

**Authors:** Marnie J. Hodge, Sara de las Heras-Saldana, Sally J. Rindfleish, Cyril P. Stephen, Sameer D. Pant

**Affiliations:** 1 Graham Centre for Agricultural Innovation (Charles Sturt University and NSW Department of Primary Industries), Charles Sturt University, Wagga Wagga, NSW 2678, Australia; mhodge@csu.edu.au (M.J.H.); cstephen@csu.edu.au (C.P.S.); 2 Apiam Animal Health, Apiam Genetic Services, Dubbo, NSW 2830, Australia; janerindfleish@gmail.com; 3 School of Environmental and Rural Science, University of New England, Armidale, NSW 2351, Australia; sdelash2@une.edu.au

**Keywords:** spermatozoal quality, RNA-sequencing, sheep, Computer Assisted Semen Analysis (CASA)

## Abstract

Reduced reproductive efficiency results in economic losses to the Australian sheep industry. Reproductive success, particularly after artificial insemination, is dependent on a number of contributing factors on both ewe and ram sides. Despite considerable emphasis placed on characterising ewe side contributions, little emphasis has been placed on characterising ram side contributions to conception success. Over 14,000 transcripts are in spermatozoa of other species, which are transferred to the ova on fertilisation. These transcripts conceivably influence early embryonic development and whether conception is successful. Semen was collected (*n* = 45) across three breeds; Merino, Dohne, and Poll Dorset. Following collection, each ejaculate was split in two; an aliquot was assessed utilising Computer Assisted Semen Analysis (CASA) and the remaining was utilised for RNA extraction and subsequent next-generation sequencing. Overall, 754 differentially expressed genes were identified in breed contrasts and contrast between ejaculates of different quality. Downstream analysis indicated that these genes could play significant roles in a broad range of physiological functions, including maintenance of spermatogenesis, fertilisation, conception, embryonic development, and offspring production performance. Overall results provide evidence that the spermatozoal transcriptome could be a crucial contributing factor in improving reproductive performance as well as in the overall productivity and profitability of sheep industries.

## 1. Introduction

Of the 45 million breeding ewes in Australia, over 250,000 are artificially inseminated each year [1]. The increasingly widespread use of artificial insemination (AI) is driven primarily by the advantages it offers over natural breeding, including the ease in use of alternative genetics, shortened lambing period, and increased number of ewes inseminated to one ejaculate. Successful conception in these assisted reproductive programs is crucial for profitable sheep farming. Low conception rates increase generation interval and reduce the number of lambs born and genetic gain, thereby having a negative impact on farm profitability. Moreover, large-scale assisted reproductive programs are time consuming, labour intensive and expensive; poor conception outcomes in these AI programs can also significantly impact farm profitability.

Reproductive success, meaning a ewe found to be pregnant following ultrasound scanning particularly after artificial insemination (AI), is dependent on a number of contributing factors on both the ewe and ram sides. While there has been considerable emphasis on characterising ewe side contributions to conception success (e.g., age, parity, etc.), relatively little emphasis has been placed on characterising ram side contributors. Literature suggests that failures during capacitation, fertilisation, or embryogenesis can all be of seminal origin [2]. Moreover, specifically in the case of sheep, conception outcomes can vary considerably between sires, with semen from some sires yielding consistently high conception rates in contrast to other sires that yield either consistently poor or highly variable conception outcomes. Since a single ejaculate from a ram has the ability to inseminate hundreds of ewes through AI programs [1], it is crucial to understand the mechanisms underlying these ram side factors that contribute to successful conception. Establishment and standardisation of accurate in vitro measures of the reproductive success of a ram are vital both prior to cryopreservation and use in artificial breeding programs. 

Historically, the motility and morphology of semen ejaculates are assessed visually via a microscope [3] prior to AI, in order to avoid the use of poor quality semen in AI programs that could lead to poor conception outcomes. While this suggests a relationship between semen quality parameters and conception outcomes, the magnitude of influence that semen quality parameters have on conception outcomes has been difficult to characterise. This is primarily because visual assessment bias between technicians has been reported to vary between 10–80% [4], which means that semen quality determined via visual assessment can be highly subjective. However, in recent decades, Computer Assisted Semen Analysis (CASA) has become possible, which enables objective, repeatable assessment of semen quality parameters. This creates an opportunity to not only objectively characterise high and low quality semen samples, but also investigate the determinants of semen quality.

One of the key factors with respect to semen quality and seminal factors that contribute to conception outcomes relates to the influence of spermatozoal RNA and the physiological role of RNA in spermatozoal cells. There are several hypotheses in the literature which range from spermatozoal RNA influencing fertilisation [5], promoting embryonic development [6], and offspring phenotype [7]. Accordingly, several studies in both animals (e.g., cattle [8], pigs [9], horses [10]); and humans [11] have attempted to characterise the spermatozoal transcriptome profiles. A previous study in pigs has also shown that spermatozoal transcript profiles can vary between high and low quality ejaculates and between breeds [9]. If similar differences exist between breeds and between high and low quality ejaculates in sheep, then this could potentially be an important determinant of successful conception and farm profitability. However, only a few studies have attempted to characterise the ovine spermatozoal transcriptome, with previous research focused on expression of protein hormone adiponectin on motility parameters [12], influence of pentose phosphate pathway enzymes on ram semen capacitation [13], and expression of sperm transcriptome following heat stress [14]. Therefore, the objective of the current study is to characterise the transcriptome of three sheep breeds common to Australia and determine whether spermatozoal transcript profiles vary between breeds and between ejaculates of varying quality. 

## 2. Materials and Methods 

### 2.1. Semen Collection 

Semen samples (*n* = 45) were collected via electro-ejaculation from three breeds of sheep; Merino (*n* = 16), Dohne (*n* = 16), and Poll Dorset (*n* = 13). Semen collection procedures were approved by Charles Sturt University under the Animal Care and Ethics Committee (approval number 19213) and consent forms were collected from commercial stud producers prior to sampling. Semen collection followed standard procedures, specified in Charles Sturt University SOP015, which aligns with defined protocols for electro-ejaculation in literature [15,16]. Rams were closely matched for age (~18 months old) and management conditions (paddock reared, native pasture grazing), all of which were located within an 80km radius of Dubbo, New South Wales. Semen collection occurred during October 2019. 

### 2.2. Semen Assessment 

#### 2.2.1. Visual Assessment Immediately Following Collection 

The ejaculate was immediately assessed for volume following collection as per the protocol described by [17], by sighting the level of semen in a graduated test tube utilised for collection. Two aliquots were obtained from the ejaculate; 250 μL was diluted 1:10 with extender (Nutrixcell) for objective assessment utilising a CASA machine, and the remaining semen was snap frozen and stored in liquid nitrogen until further RNA isolation.

#### 2.2.2. Computer Assisted Semen Assessment 

In order for objective assessment of each ejaculate to be performed, the diluted aliquot of 250 μL of fresh semen was maintained at 37 °C in a temperature regulated water bath. At 4 h post collection, Computer Assisted Semen Analysis (CASA) was performed to objectively and accurately assess semen quality [18]. CASA was performed using the Hamilton Thorne IVOS II (Hamilton Thorne, MA, USA) by preparing samples according to the manufacturer’s instructions. The IVOSS II VIADENT software with a frame rate of 60 Hz/s was used to capture 60–80 sperm per field of view across 6–8 total fields of view, capturing approximately 400 spermatozoa for each sample analysis. In addition to these kinematics, sperm concentration, percentage motile sperm, and percent abnormal sperm were determined via CASA. Ranking of ejaculate quality followed semen assessment, and was based on a previously described procedure [19] that relies on consideration of a broad range of semen quality parameters including percentage of motility, straight line velocity (VSL), curvilinear velocity (VCL), average path velocity (VAP), and percentage of morphologically normal spermatozoa.

### 2.3. RNA Isolation 

Prior to RNA isolation, the volume of semen required for an adequate RNA yield was determined according to the previously described protocol [20]. RNA isolation was performed using the RNeasy Plus Universal Mini Kit (Qiagen, Hilden, Germany). Following RNA isolation, the RNA integrity was assessed with a NanoPhotometer Spectrophotometer (Implen, CA, USA). The RNA integrity number (RIN) was assessed and samples with a RIN higher than 8 were kept for sequencing. After quality control of RNA samples, a total of 36 samples, (i.e., 12 samples each of Merino, Dohne, and Poll Dorset breeds); were chosen for RNA sequencing. 

### 2.4. Library Preparation and Sequencing 

The NEBNext Ultra RNA Library Prep Kit for Illumina (New England Biolabs, Ipswich, MA, USA) was used to fragment the RNA and synthesise the complementary DNA (cDNA) library following the manufacturer’s instructions. The sequencing of the cDNA libraries was performed with the Illumina Hiseq2000 platform, obtaining 100 bp paired-end reads (PE). 

### 2.5. Bioinformatics Analysis 

Following sequencing, the quality of reads was assessed with FastQC v0.11.5 (https://www.bioinformatics.babraham.ac.uk/projects/fastqc/) (Bioinformatics). Poor quality bases (Phred score Q < 30), adaptors, and overrepresented sequences were filtered out with trimmomatic v0.36 [21]. The sheep reference genome *Ovis* aries (Oar v.4.) was used to map the reads with TopHat v2.1.1 [22]. Reads that were mapped to only one location were kept and annotated to the reference genome to identify the gene counts using HTSeq v0.6.1 [23]. Further quality control was applied to exclude outliers and samples with low mapping rates to the ovine genome. Consequently, the final cohort for differential gene expression analysis included 9 Merino, 10 Dohne, and 12 Poll Dorsets. 

### 2.6. Differential Expression Analysis 

Quality control was performed on the gene counts and log count per millions (CPM) to remove the genes with low expression (≤10 counts in a library) using edgeR package v3.6.1 [24]. Differential gene expression analysis was performed within the R software environment using the DESeq 2 package v1.24 [25] to identify all genes that were either up- or down-regulated with a log fold change (FC) >1. A false discovery rate threshold of (FDR) < 0.05 was applied to control type I error. In total, Merino (*n* = 9), Dohne (*n* = 10), and Poll Dorset (*n* = 12) were utilised for differential expression analysis. Four contrasts were performed, out of which three were between breeds (Dohne vs. Merino, Dohne vs. Poll Dorset, and Merino vs. Poll Dorset); and one contrast was performed to compare ejaculates of relatively high and low qualities fitting the breeds, to account for possible breed differences. The R functions Venn Diagram v1.6.20 [26] and heatmap.2 [27] were used to draw the Venn diagram and the heat map respectively. A functional analysis was performed with the Cytoscape plug-in ClueGo [28] to gain insight into the enriched pathways and gene ontology terms. 

## 3. Results

### 3.1. Quality of Ejaculate 

The quality of each ejaculate was assessed both subjectively for volume and objectively by a CASA machine for semen quality parameters. Table 1 displays average descriptive statistics for semen parameters for Merino, Dohne, and Poll Dorset rams. 

### 3.2. Gene Expression 

The descriptive statistics shown in Table 2 summarise the yields of raw, cleaned, and mapped reads, along with the mapped rate (%) following RNA sequencing. A total of 1,187,335,440 mapped reads across the three breeds sampled were mapped against the latest publicly available reference genome (*O. aries)* at a rate of 88%. A complete summary of cleaned and mapped reads is provided in Supplementary Table S1.

### 3.3. Differentially Expressed Genes (DEG) Analysis and Functional Analysis

Table 3 displays the total number of differentially expressed genes (DEGs) for the comparisons between breeds and the comparison of ejaculate quality across breeds. In total, 754 DEGs were identified via the four different comparisons. From these, there were 39 DEGs found when comparing various ejaculate qualities: higher quality ejaculates versus lower quality ejaculates. A complete list of DEGs is provided in Supplementary Table S2.

**Figure 1:**
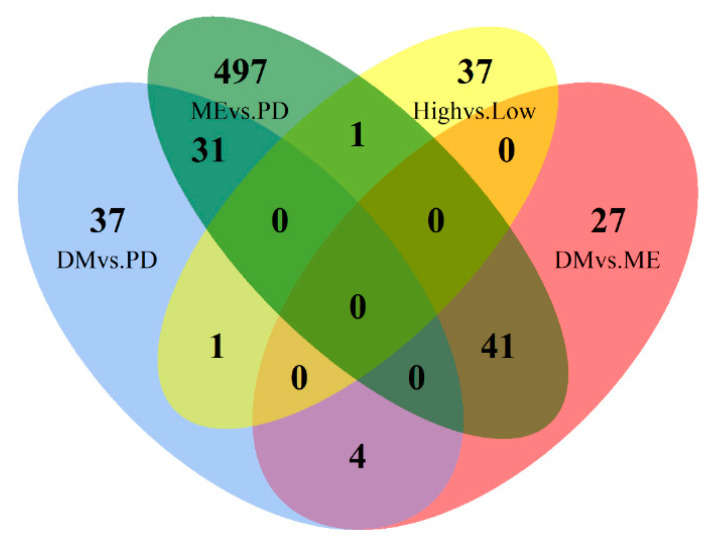
Venn diagram depicting the number of differentially expressed genes (DEGs) for each breed; DM: Dohne, PD: Poll Dorset, ME: Merino.

A number of DEGs were found to be commonly expressed within specific comparisons of breeds sampled. The Venn diagram in Figure 1 depicts the number of DEGs as well as the number of genes which are expressed within multiple breed contrasts. The Merino vs. Poll Dorset breed comparison has the largest number of DEGs, whereas the comparisons of Dohne vs. Poll Dorset and Dohne vs. Merino identified much lower, comparable DEGs.

**Figure 2:**
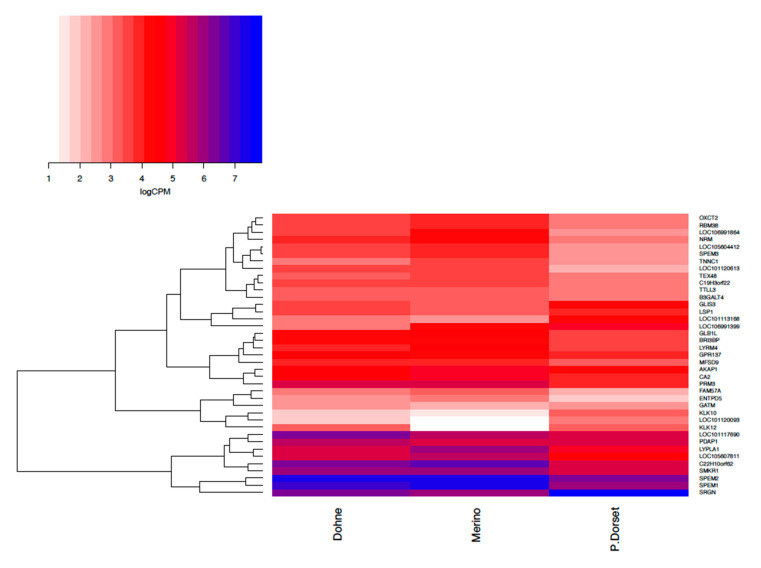
Cluster heat map comparing differentially expressed genes between sampling groups: Dohne, Merino, and Poll Dorset rams.

According to the number of differentially expressed genes between contrasts, the transcriptomic profile of the Merino and Dohne rams appear to be similar, with the spermatozoal transcriptomic profile of the Poll Dorset having more differences when compared to the Merino. This is further represented in the heat map in Figure 2, comparing the differentially expressed genes between sampling groups; Dohne, Merino, and Poll Dorset ram breeds. The top differentially expressed genes identified via the ejaculate quality contrast were selected to run a cluster analysis. Figure 2 depicts genes in white associated with a lower level of expression in comparison to the genes coloured blue, which had higher level of expression. The Poll Dorset group shows different gene expression abundances in comparison to both the Dohne and Merino rams, which have similar gene expression profiles according to the heat clustering map. 

Table 4 depicts the top 10 differentially expressed genes across the breeds, contrasting various ejaculates of relatively high and low quality. A literature search including studies in comparable livestock species [8,9,10] showed that of the top 10 DEGs between breeds, genes were identified as being either associated with fertility and embryonic development or with production performance traits.

### 3.4. Common Differentially Expressed Genes between Breed Contrasts 

A number of common DEGs between each breed contrast were found (Table 5) and are also depicted in the Venn diagram in Figure 1. A search of previous literature found that 9, 2, and 3 common DEGs for Merino, Dohne, and Poll Dorset rams respectively were associated with conception and embryonic development. Furthermore, 11 genes were each found, for Merino and Poll Dorset rams respectively, to be associated with offspring growth and carcass development. On the other hand, 4 and 8 genes were found to be linked with breed-specific production traits, corresponding to wool production and carcass performance for Merino and Poll Dorset rams respectively. 

### 3.5. Pathway Enrichment and Gene Ontology Enrichment Analysis 

According to the differentially expressed genes from the breed contrasts and ejaculate quality, a total of 36 biological processes (defence responses to other organisms, organic substance transport, cellular response to cytokine stimulus, etc.), three cellular components (vacuole, lysosome, lytic vacuole), and two molecular functions (identical protein binding, protein domain specific binding) gene ontology (GO) terms were significantly enriched (Table 6). Similarly, there was one single pathway (osteoclast differentiation) found to be significantly enriched following the pathway analysis. Detailed information on the enriched GO terms is provided in the Supplementary File Table S3. 

The top ten DEGs between Merino and Dohne rams were involved in 8 GO terms including identical protein binding, regulation of response to stimulus, regulation of defence response, inflammatory response, and defence response. Another 18 GO terms were found to be enriched by the top 10 DEGs from the Merino vs. Poll Dorset contrast, including animal organ development, tissue development, cell surface receptor signalling pathway, organic substance transport, and regulation of multicellular organismal process. Finally, 21 GO terms were found to be associated with the top 10 DEGs between the Dohne vs. Poll Dorset rams, including animal organ development, anatomical structure morphogenesis, programmed cell death, apoptotic process, and organic substance transport. 

**Figure 3:**
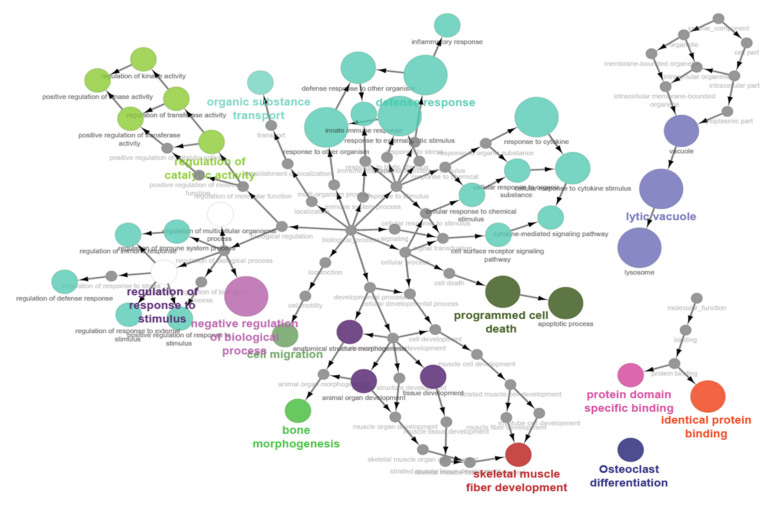
Functional network inferred from the gene ontology (GO) terms enriched in genes differentially expressed from the four contrast groups. The size of the circle indicates the number of genes associated with that GO term.

There were 7 GO terms enriched by the top 10 DEGs identified by contrasting ejaculates of varying quality, including animal organ development, tissue development and programmed cell death, and regulation of multicellular organismal process. The visualisation of the gene ontology (GO) enrichment analysis, in the form of a functional network shown in Figure 3, was also performed to identify the molecular processes and biological GO which were enriched across the 4 contrast (3 breed contrasts and ejaculate quality contrasts). 

## 4. Discussion

Spermatozoal cells contain a large repertoire of RNA transcripts that are transferred to the ovum during fertilisation. However, the physiological role of spermatozoal RNA, particularly in relation to fertility and embryonic development, remains largely unknown. Therefore, the key objectives of this study were to characterise the ovine spermatozoal transcriptome and to determine whether transcriptomic profiles varied between breeds and between semen ejaculates of varying quality. 

A total of 754 differentially expressed genes (DEGs) were identified in this study when comparing spermatozoal transcript profiles of three common sheep breeds, and the top 10 DEGs identified in each breed contrast were subjected to a literature review. Genes of interest identified in the Merino vs. Dohne contrast include *5′-Aminolevulinate Synthase 1 (ALAS1*), *Capping Protein Regulator and Myosin 1 Linked* (*CARMIL1*), and *Mevalonate Kinase* (*MVK*) (Table 4). The gene *ALAS1* is known to regulate circadian networks in cattle, which could play a crucial role in the regulation of reproduction in seasonally breeding species like sheep [31]. A bovine genome wide association study (GWAS) identified *CARMIL1* to be significantly associated with fertility [37]. Finally, *MVK* has been found to be significantly associated with regulation of cholesterol synthesis [69], which in turn, is thought to play a significant role in spermatogenesis [70]. 

In the Merino vs. Poll Dorset contrast, *Solute Carrier Family 35 Member A5* (*SLC35A5*) and *Integral Membrane Protein 2C* (*ITM2C*) were identified as key DEGs, both of which have been reported for their association with conception and embryonic development [40,44] (Table 4). Specifically, in cattle, a GWAS found *SLC35A5* to be associated with fertility [40]; while the precise physiological role of *ITM2C* is not yet known, expression of *ITM2C* is significantly enriched in the epididymis and vas deferens in both humans and mice during sexual maturation [46]. Lastly, key DEGs identified in the Dohne vs. Poll Dorset contrast included *DNA Polymerase Kappa* (*POLK*)*,* which is developmentally regulated in the testis of human and mice, and is hypothesised to play a significant role in [55]; and *Mannosidase Alpha Class 1A Member 1 (MAN1A1*), which has been reported to be associated with weight at 6 months in sheep in a GWAS [57] (Table 4).

There have been limited investigations aimed at characterising the spermatozoal transcriptomes of different breeds in livestock species. However, a previous study focused on pigs did report breed-specific differences in spermatozoal transcript profiles, which is similar to the findings in this study [9]. Specifically, this study reported that Durocs differed significantly when compared to the Yorkshire and Landrace breeds, and ultimately suggested that the conception, growth, and offspring phenotype may be significantly influenced by spermatozoal transcripts of the pigs [9]. Overall, results from both the current study and the previous porcine study indicate that breed-specific differences in spermatozoal transcriptome profiles exist, and the physiological relevance of these differences, particularly with respect to animal production, warrants further investigation. 

When contrasting ejaculates that were determined to be relatively high and low quality, the key DEGs identified included *SPEM Family Member 2* (*SPEM2*), *3-Oxoacid CoA-Transferase 2* (*OXCT2*), and *Family with Sequence Similarity 57 Member A* (*FAM57A*). In a GWAS in pigs, *SPEM2* was found to be associated with embryonic development and number of piglets born alive [60]. Georgiadis et al. [65] used *OXCT2* as a post-fertilisation and early embryonic marker using quantitative polymerase chain reaction (qPCR) when investigating high quality RNA in human semen. Similarly, *FAM57A* has been shown to be upregulated in meiosis in mice, and is thought to play a crucial role in spermatogenesis [67]. 

Results from gene set enrichment analyses further identified several biological pathways that accord with the results observed in differential gene expression analyses. The top pathways found to be significantly enriched in sampling groups included cell migration, regulation of molecular function, bone morphogenesis, organic substance transport, apoptotic processes, skeletal muscle fibre development, and animal organ development. It is noteworthy that a previous study in horses, which aimed to contrast the spermatozoal transcriptomes of stallions with differing conception rates, reported significantly enriched biological pathways similar to those identified in this study, including cellular function and maintenance, cellular growth and proliferation, cellular movement, cell death and survival, tissue morphology, and organ development [10]. Given that a lot of these pathways would be relevant to embryonic development, these results indicate that spermatozoal transcripts could play a role in embryonic development and thereby contribute to either successful conception, or possibly the maintenance of pregnancy in animals. 

Comparison of the differentially expressed genes from one breed with the other two breeds indicated unique expression profiles. 41, 4, and 31 genes were found to be expressed at a significantly different level when compared to the other two breeds for Merino, Dohne, and Poll Dorset, respectively (Table 5), suggesting that each gene has a unique expression profile in said breed. 

In total, 41 DEGs were identified in common, when contrasting Merinos with either Poll Dorsets or Dohnes. Of these, 9 have been found, in the literature, to significantly influence reproductive output and embryonic development. Specifically, *Mitogen-Activated Protein Kinase 3* (*MAPK3*), *Mediator Complex Subunit 6* (*MED6*), *WAS/WASL-Interacting Protein Family Member 2* (*GTL2*)*,* and *Spectrin Beta, Non-Erythocytic 2* (*SPTBN2*) have been reported for associations with in vitro oocyte maturation [71], stem cell development [36], normal embryonic development [72], and cytoskeletal development [73], respectively. Similarly, *Mevalonate Kinase* (*MVK*) [69] and *Chondroitin Sulfate N-Acetylgalactosaminyltransferase 1* (*CSGALNACT1*) [74] have been linked with regulation of cholesterol synthesis and copper metabolism respectively, both of which are thought to play crucial roles in spermatogenesis and reproductive performance. Finally, *Tensin 3* (*TNS3*) is known to be expressed in the endometrium during parturition in pigs [30], and *Obscurin Like Cyotoskeletal Adaptor 1* (*OBSL1*) is significantly expressed in ovarian follicles in cattle [75], which could indicate a physiological role in either conception or embryonic development. 

Of the 41 DEGs identified in common when contrasting Merinos with either Poll Dorsets or Dohnes, nine of those genes have been associated with growth and development following a literature search. Previous studies show that genes *Muskelin* (*MKLN1*) [76], *Carbohydrate Sulfotransferase 4* (*CHST4*) [77], *Family with Sequence Similarity 210 Member B* (*FAM210B*) [44], and *Limb Development Membrane Protein 1* (*LMBR1*) [78] have been shown to be respectively associated with back fat accumulation in pigs, enriched in pathways associated with feed intake in cattle, meat quality in pigs, and normal limb and embryonic development in both chickens and pigs, suggesting roles in growth and development of offspring. The following common DEGs for Merino rams are associated with breed specific production performance traits: *Transforming Growth Factor Beta 2* (*TGFB2)* [79], *Keratin 4* (*KRT4*) [80], and *Involucrin* (*IVL)* [81]. These have all been found in the literature to be expressed during skin tissue and wool follicle development. Similarly, *Intersectin* 1 (*ITSN1*) is directly associated with the polled trait in cattle [82], suggesting it is significant in the Poll Merino ram phenotype, as this gene was upregulated for the Merino when contrasting Merino and Poll Dorset rams. 

Four genes identified in common when contrasting Dohnes with either Merino or Poll Dorsets have been found in the literature to have various influences on growth and development: *Capping Protein Regulator and Myosin 1 Linker 1* (*CARMIL1*)*, Fibronectin Leucine Rich Transmembrane Protein 2* (*FLRT2*), *ATP Binding Cassette Subfamily A Member 4* (*ABCA4*), and *Zinc Finger Protein 382* (*ZNF382)*. Cole et al. [38] found that a SNP located close to *FLRT2* is associated with significant effects on embryonic development and live birth weight in cattle. Similarly, previous studies have highlighted the importance of *ABCA4, ZNF382,* and *CARMIL1* in the development of neural tissue [83], heart development, and function [84], and the importance of ensuring motility of cells, such as lamellipodium, which is important for cell survival [37]. 

Of the 31 DEGs found in common when contrasting Poll Dorset rams with either Merinos or Dohnes, 11 were found to be associated with growth and development, 3 with fertility and reproduction, and 8 with carcass performance. *Peptidase Domain Containing Associated With Muscle Regeneration 1* (*PAMR1*) and *Sorbin and SH3 Domain Containing 2* (*SORBS2*) have been found to respectively influence formation and development of skin [49], and muscle tissue and disposition of marbling (IMF) in cattle [85]. Puttabyatappa [58] found *Chintase 3 Like 1 (CHI3L1*) to be differentially expressed and play a role in sheep follicular function, suggesting an importance in embryonic development. Similarly, *Lymphocyte Antigen 6 Family Member E* (*LY6E*) [50] has been discovered to be significantly expressed in biological pathways associated with embryonic development in cattle. The *Ring Finger Protein 151* (*RNF151*) gene has been found to be exclusively expressed in the testis and plays a crucial role in the spermatogenesis, and as such is crucial for the regulation of male reproductive performance [86,87]. 

Previous studies emphasise the findings of this current study, with common DEGs between each sampling breed suggesting that the spermatozoal transcriptome plays an important role in conception and embryonic development. 

## 5. Conclusions

The spermatozoal transcriptomes of sheep seem to be specific and include a large repertoire of genes that have been reported to play crucial roles in reproduction. Spermatozoal transcript profiles seem to differ between breeds in sheep, and this could have some relevance to either reproduction outcomes in these breeds, and even for embryonic development. In fact, if embryonic development is significantly influenced by spermatozoal transcripts, then it is possible that postnatal phenotypes (e.g., birth weight, weight gain, weaning weight, etc.) are also influenced, at least in part, by the spermatozoal RNA that is contributed to the zygote. Therefore, the current study provides important insights into spermatozoal transcriptomes in sheep and suggests that further investigation delving into the physiological role of spermatozoal transcripts is warranted. Such investigations could help in understanding the mechanisms that underlie successful reproduction outcomes in sheep, and possibly other livestock species, given that reproductive physiology is generally conserved across mammalian species. Moreover, these investigations could lead to the development of molecular markers and in vitro measures that could assist in predicting successful reproduction when specific ejaculates are used in artificial breeding programs.

## Figures and Tables

**Table 1:** Semen parameters (Mean ± SE) assessed via Computer Assisted Semen Analysis (CASA) for Dohne, Merino, and Poll Dorset.

Assessment Parameters	Merino	Dohne	Poll Dorset
Motility	64.25 ± 6.69	75.99 ± 4.30	74.62 ± 3.86
VSL (μm/s)	110.51 ± 11.30	105.85 ± 8.18	125.85 ± 11.73
VCL (μm/s)	278.86 ± 27.16	238.71 ± 22.95	277.10 ± 23.11
VAP (μm/s)	10.17 ± 1.00	9.15 ± 1.04	10.87 ± 1.05
ALH (μm/s)	149.93 ± 12.78	139.18 ± 10.08	161.65 ± 11.58
LIN	43.50 ± 3.63	37.93 ± 3.89	51.19 ± 4.93
STR (%)	80.00 ± 2.49	57.69 ± 3.64	75.57 ± 3.44
WOB	54.55 ± 2.92	47.86 ± 3.42	57.55 ± 3.69
BCF (Hz)	36.35 ± 1.21	26.67 ± 2.28	33.34 ± 1.74
Membrane Integrity (%)	89.18 ± 2.40	82.44 ± 2.61	91.54 ± 2.80
Morphologically Normal (%)	83.51 ± 3.31	96.18 ± 0.43	89.28 ± 1.79

Note: VLS: straight line velocity; VCL: curvilinear velocity; VAP: average path velocity; ALH: amplitude of lateral head displacement; LIN: linearity; STR: straightness; WOB: wobble; and BCF: beat cross frequency.

**Table 2:** Summary of reads quality and mapping in RNA sequencing analysis.

	Total Raw Reads	Total Clean Reads	Mapped Reads	Mapped Rate %
**Merino**	564,403,692	533,122,904	693,063,473	85.8%
**Dohne**	548,882,390	517,471,028	222,273,782	89.3%
**Poll Dorset**	683,941,522	643,781,418	271,998,185	89.0%
**Total**	1,797,227,604	1,694,375,350	1,187,335,440	88.0%

**Table 3:** Numbers of differentially expressed genes for the comparison of treatment groups.

	Differentially Expressed Genes	Upregulated	Downregulated
**Dohne vs. Merino**	72	25	47
**Dohne vs. Poll Dorset**	73	41	31
**Merino vs. Poll Dorset**	570	234	336
**Ejaculate Quality Contrasts**	39	6	33

**Table 4:** List of top 10 differentially expressed genes across the three breeds sampled (Merino, Dohne, and Poll Dorset) and ejaculates of varying quality.

Gene	logFC	Reference
**Dohne vs. Merino**		
*Mevalonate Kinase* (MVK) ^A^	2.16	[29]
*Tensin 3* (TNS3) ^A^	−1.82	[30]
*5′-Aminolevulinate Synthase 1* (ALAS1) ^A^	1.31	[31]
*Cellular Communication Network Factor 1* (CCN1) ^A^	−2.17	[32]
*Methionine Sulfoxide Reductase A* (MSRA) ^A^	2.33	[33]
*Ribosomal RNA Processing 15 Homolog* (RRP15) ^A^	−2.57	[34]
*Peroxisome Proliferator Activated Delta* (PPARD) ^B^	1.47	[35]
*Mediator Complex Subunit 6* (MED6) ^A^	−1.35	[36]
*Capping Protein Regulator And Myosin 1 Linked* (CARMIL1) ^A^	1.26	[37]
*Fibronectin Leucine Rich Transmembrane Protein 2* (FLRT2) ^A^	−2.08	[38]
**Merino vs. Poll Dorset**		
*ATPase Sarcoplasmic/Endoplasmic Reticulum Ca^2+^ Transporting 3* (ATP2A3) ^A^	−2.59	[39]
*Solute Carrier Family 35 Member A5* (SLC35A5) ^A^	1.12	[40]
*BLOC-1 Related Complex Subunit 5* (BORCS5) ^A^	2.08	[41]
*Methionine Sulfoxide Reductase A* (MSRA) ^B^	−2.64	[42]
*Keratin 4* (KRT4) ^B^	−1.78	[43]
*Family With Sequence Similarity 210 Member B* (FAM210B) ^B^	−1.47	[44]
*NTPase KAP Family P-Loop Domain Containing 1* (NKPD1) ^B^	2.89	[45]
*Integral Membrane Protein 2C* (ITM2C) ^A^	−3.05	[46]
*Annexin A2* (ANAX2) ^A^	−1.46	[47]
*Solute Carrier Family 2 Member 3* (SLC2A3) ^A^	−2.98	[48]
**Dohne vs. Poll Dorset**		
*Peptidase Domain Containing Associated With Muscle Regeneration 1* (PAMR1) ^B^	−2.56	[49]
*Lymphocyte Antigen 6 Family Member E* (LY6E) ^A^	−1.33	[50]
*Arginine And Glutamate Rich 1* (ARGLU1) ^B^	1.64	[51]
*Cysteine Dioxygenase Type 1* (CDO1) ^B^	2.10	[52]
*Valyl-TRNA Synthetase 2, Mitochondrial* (VARS2) ^A^	−2.13	[53]
*Tumor Protein P53 Inducible Protein 11* (TP53I11) ^A^	2.15	[54]
*DNA Polymerase Kappa* (POLK) ^A^	1.65	[55]
*F-Box And Leucine Rich Repeat Protein 14* (FBXL14) ^B^	−1.25	[56]
*Mannosidase Alpha Class 1A Member 1* (MAN1A1) ^A^	−2.26	[57]
*Chintase 3 Like 1* (CHI3L1) ^A^	−2.29	[58]
**Ejaculate Quality Contrast**		
*Kallikrein Related Peptidase 12* (KLK12) ^B^	2.65	[59]
*SPEM Family Member 2* (SPEM2) ^A^	−2.46	[60]
*Lysophospholipase 1* (LYPLA1) ^A^	−2.31	[61]
*Troponin C1, Slow Skeletal And Cardiac Type* (TNNC1) ^B^	−2.23	[62]
*LYR Motif Containing 4* (LYRM4) ^B^	−1.97	[63]
*Major Facilitator Superfamily Domain Containing 9* (MFSD9) ^A^	−1.63	[64]
*3-Oxoacid CoA-Transferase 2* (OXCT2) ^A^	−1.88	[65]
*Small Lysine Rich Protein 1* (SMKR1) ^B^	−1.96	[66]
*Family With Sequence Similarity 57 Member A* (FAM57A) ^A^	−1.72	[67]
*Serglycin* (SRGN) ^A^	2.38	[68]

Note: The genes with a superscript ^A^ are associated with fertility and embryonic development and genes with superscript ^B^ are associated with production performance and carcass traits; logFC: log fold change.

**Table 5:** List of common differentially expressed genes which were significantly expressed for each comparison group (seen in Figure 1); Merino, Dohne, and Poll Dorset rams.

Comparison Groups	Number of Common Differentially Expressed Genes	Common Differentially Expressed Genes
**Merino vs. (Poll Dorset and Dohne)**	41	*RGCC, MVK ^A^, LOC101101919, LOC101121185, TNS3 ^A^, NFYB, MSRA, ZNF706, RRP15, PPARD, MED6 ^A^, LOC101102105, LOC105612466, LMBR1 ^B^, TGFB2 ^C^, ARHGDIA ^B^, MAPK3 ^A^, DHX40 ^B^, LOC114114020, MKLN1 ^B^, PITX1 ^B^, CHST4 ^B^, FCAMR ^B^, CSGALNACT1 ^A^, COMMD3 ^A^, SFN, LOC114115624, RIC8A, PRR14L ^A^, CHPF, BORCS5, LOC101103973, KRT4 ^C^, ITSN1 ^C^, IVL ^C^, GTL2 ^A^, FAM210B ^B^, SPTBN2 ^A^, DEAF1 ^B^, LOC114110979, OBSL1 ^A^*
**Dohne vs. (Poll Dorset and Merino)**	4	*CARMIL1 ^A^, FLRT2 ^A^, ABCA4, ZNF382*
**Poll Dorset vs. (Dohne and Merino)**	31	*LOC101108654, PAMR1 ^B^, LY6E ^B^, ARGLU1 ^C^, LOC101113735, CDO1 ^C^, TP53I11, MAN1A1 ^∇^, CHI3L1 ^A^, GAA, SORBS2 ^B^, APOM ^C^, BPIFB1, HEMK1 ^C^, LPO, CDH26 ^B^, NUAK1, UNC5B ^A^, SGSH ^B^, VGF ^C^, GSDMA ^C^, FCGR3A ^B^, HSPB8 ^B^, RNF151 ^A^, AFAP1L2 ^B^, ANKEF1, SLC48A1 ^C^, DHRS3 ^B^, G0S2 ^B^, UBXN8 ^B^, LOC101103862*

Note: The genes with superscript ^A^ are associated with fertility and embryonic development; genes with superscript ^B^ are associated with growth and development; and genes with superscript ^C^ are associated with breed specific production performance.

**Table 6:** Top 10 Gene Ontology (GO) terms for molecular functions, cellular components, and biological processes of spermatozoal transcripts across Merino, Dohne, and Poll Dorset.

GO Term	*p*-Value	Number of Genes
**Molecular Functions**		
Identical protein binding	2.16 × 10^−5^	64
Protein domain specific binding	1.05 × 10^−4^	28
**Cellular Components**		
Vacuole	9.96 × 10^−6^	24
Lysosome	9.96 × 10^−6^	22
Lytic vacuole	9.96 × 10^−6^	22
**Biological Processes**		
Defence response to other organism	4.98 × 10^−8^	34
Cellular response to cytokine stimulus	4.98 × 10^−8^	35
Cellular response to organic substance	4.98 × 10^−8^	67
Cellular response to chemical stimulus	4.98 × 10^−8^	81
Response to other organism	4.98 × 10^−8^	49
Regulation of multicellular organismal process	4.98 × 10^−8^	89
Regulation of immune response	4.98 × 10^−8^	30
Positive regulation of response to stimulus	4.98 × 10^−8^	67
Regulation of response to stimulus	4.98 × 10^−8^	107
Innate immune response	4.98 × 10^−8^	24

## Data Availability

The data presented in this study are available on request from the corresponding author.

## Supplementary Materials

The following are available online at https://www.mdpi.com/2073-4425/12/2/203/s1, Table S1. Summary of the cleaning and mapping of the reads, Table S2. A complete list of DEGs, Table S3. Detailed information on the enriched GO terms.
